# Asthma diagnosis and treatment - 1027. Body mass index of asthmatic children in United Arab Emirates

**DOI:** 10.1186/1939-4551-6-S1-P26

**Published:** 2013-04-23

**Authors:** Suleiman Al-Hammadi, Olfat Al-Zaabi

**Affiliations:** 1 Pediatrics, College of Medicine and Health Sciences, UAE University, Al-Ain, United Arab Emirates; 2 Pediatrics, Fujairah Hospital, Fujairah, United Arab Emirates

## Background

Obesity is common, especially in children with asthma. An entity termed “childhood obesity-associated asthma” was proposed to be linked to Th1 polarization rather than the common Th2 profile of allergic diseases.

In one study, high body mass index (obesity or overweight) was associated with asthma at all ages. Another study showed obesity negatively impacted asthma control. In a Japanese study, the cutoff for BMI prediction of asthma was lower than the Westerns. Consistently, race and ethnicity were shown to influence the association between obesity and asthma.

The aim of this study was to explore whether Emeriti asthmatic children have higher body mass index (BMI).

## Methods

This cross-sectional study involved public school children (age = 12 to16 years in the 7^th^ and 8^th^ grades) in the Eastern province of United Arab Emirates (UAE). A questionnaire (21 exploring questions to be answered by the parents) was given to 1,218 students of the chosen classes. Eight hundred ninety one (73%) students retuned the survey and were included in the study. Other collected data included students’ height, weight, FEV1 and PEF.

## Results

The prevalence of asthma was 13% (115 of 891 students). There were no difference in the asthma prevalence among males and female (*p*=0.159). BMI (mean ± SD) for the asthmatics was 21.43 ± 7.01 and non-asthmatics 20.22 ± 5.53 (*p*=0.076). Atopic dermatitis (*p*=0.001), allergic rhinitis (*p*<0.001) and food allergy (*p*<0.05) were higher among the asthmatics.

## Conclusions

BMI was higher among asthmatics, although the difference did not reach a statistical significance. Atopic disorders significantly clustered in asthmatic children.

